# SemFunSim: A New Method for Measuring Disease Similarity by Integrating Semantic and Gene Functional Association

**DOI:** 10.1371/journal.pone.0099415

**Published:** 2014-06-16

**Authors:** Liang Cheng, Jie Li, Peng Ju, Jiajie Peng, Yadong Wang

**Affiliations:** 1 Center for Bioinformatics, School of Computer Science and Technology, Harbin Institute of Technology, Harbin, Heilongjiang, China; 2 School of Electrical and Electronic Engineering, Nanyang Technological University, Singapore, Singapore; University of Turin, Italy

## Abstract

**Background:**

Measuring similarity between diseases plays an important role in disease-related molecular function research. Functional associations between disease-related genes and semantic associations between diseases are often used to identify pairs of similar diseases from different perspectives. Currently, it is still a challenge to exploit both of them to calculate disease similarity. Therefore, a new method (SemFunSim) that integrates semantic and functional association is proposed to address the issue.

**Methods:**

SemFunSim is designed as follows. First of all, FunSim (Functional similarity) is proposed to calculate disease similarity using disease-related gene sets in a weighted network of human gene function. Next, SemSim (Semantic Similarity) is devised to calculate disease similarity using the relationship between two diseases from Disease Ontology. Finally, FunSim and SemSim are integrated to measure disease similarity.

**Results:**

The high average AUC (area under the receiver operating characteristic curve) (96.37%) shows that SemFunSim achieves a high true positive rate and a low false positive rate. 79 of the top 100 pairs of similar diseases identified by SemFunSim are annotated in the Comparative Toxicogenomics Database (CTD) as being targeted by the same therapeutic compounds, while other methods we compared could identify 35 or less such pairs among the top 100. Moreover, when using our method on diseases without annotated compounds in CTD, we could confirm many of our predicted candidate compounds from literature. This indicates that SemFunSim is an effective method for drug repositioning.

## Background

The quantitative measurement of similarity between diseases based on qualitative association [1]–[5] raises more and more attention, because it plays an important role in predicting disease-causing genes [6], [7], inferring microRNA function associations [8], and identifying novel drug indications [9]. Currently, there is a critical need to design methods to measure disease similarity.

Methods for calculating disease similarity can be broadly classified as semantic-based [8], [10] and function-based [11]–[13]. Semantic-based methods are widely used for measuring similarity between terms of Gene Ontology (GO) [14], [15] and human phenotype ontology (HPO) [16] in the biomedical and bioinformatics domain. Few of them are used for calculating similarity between terms of disease-related ontologies. For computing the similarity of GO terms, Resnik's method [17] has a better performance evaluation result [18] than union-intersection (UI), longest shared path (LP), JC [19] and Lin [20]. Resnik's method has also been used to calculate the similarity between terms of Disease Ontology (DO) [10], [21], measuring disease similarity based on the information content (IC) (Figure S1 and File S1) of the most informative common ancestor (MICA) (Figure S1 and File S1) between two terms. In addition, Wang et al.'s method [22] calculates similarity between terms considering multiple common ancestors. It performs very well for computing the semantic similarity between GO terms [22], and has been successfully used for measuring disease similarity between medical subject headings (MeSH) [23] terms and inferring microRNA function network [8].

Function-based methods calculate disease similarity by comparing disease-related gene sets [11]–[13]. Mathur and Dinakarpandian [11] designed the similarity method based on overlapping gene sets (BOG) between diseases of DO. In comparison to semantic-based methods, the BOG method defines disease similarity from a new perspective. Therefore, it is possible to find unknown relationships [11]. However, it ignores the functional associations between disease-related genes which contribute to disease similarity. In another method, Mathur et al. [13] presented a process-similarity based (PSB) method by involving the associations based on GO [14] terms. PSB outshines BOG, and its performance is better than Resnik [17], Lin [20], LC [24] and JC's [19] methods [13]. Functional associations between genes involve multiple aspects, such as co-expression [25], protein-protein interaction [26], GO terms [27], etc. However, the PSB method only exploits the associations from GO terms. Therefore, the performance would likely be better if multiple associations were considered for calculating disease similarity.

**Figure 1:**
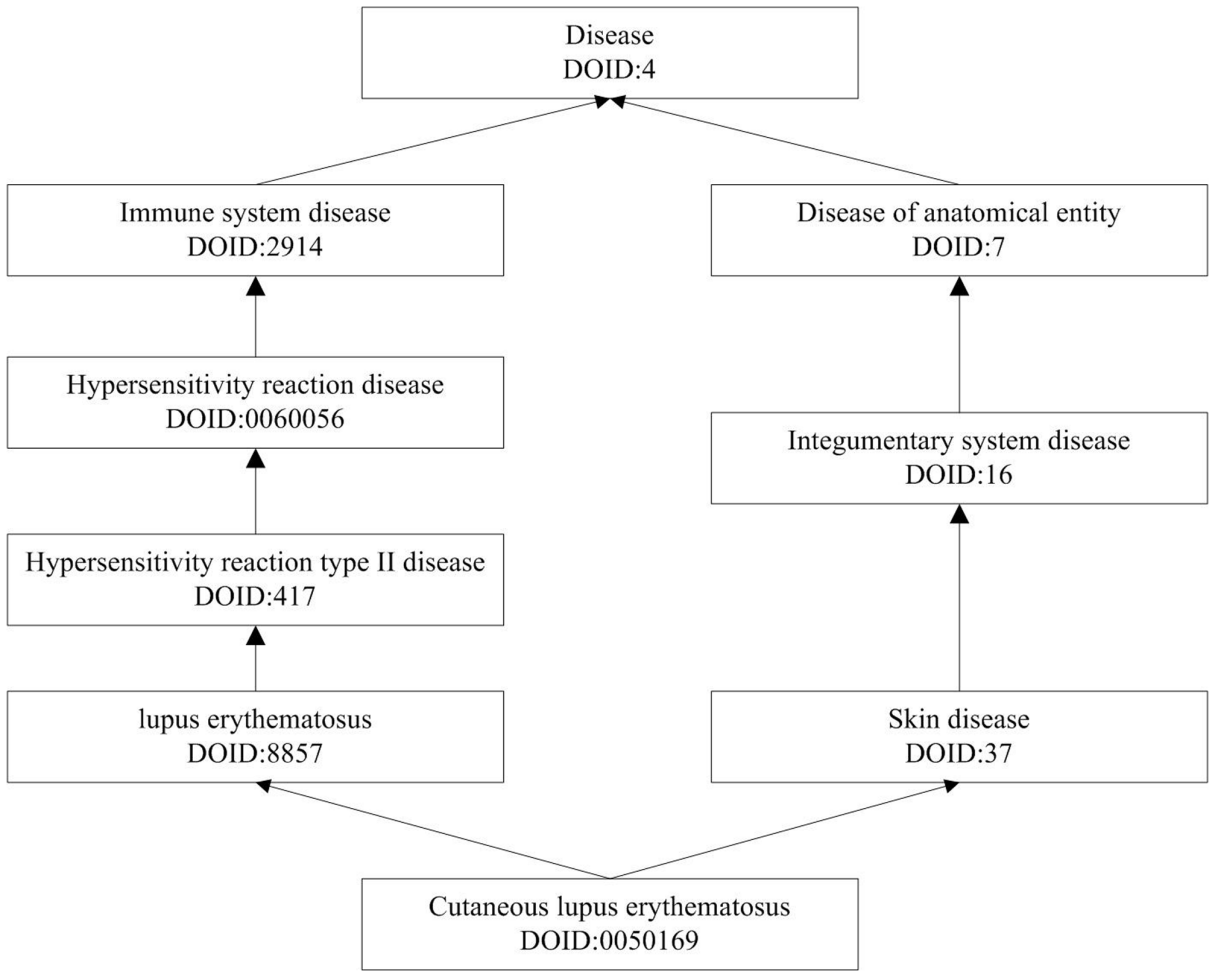
A sub-graph of the DAG for DO term ‘Cutaneous lupus erythematosus (DOID:0050169)’. The arrow symbol represents an ‘IS_A’ link of DO. For example, “Cutaneous lupus erythematosus (DOID:0050169)” is linked to “Skin disease (DOID:37)” by an ‘IS_A’ relationship.

There are many disease-related vocabularies, some of which describe semantic associations between diseases by ‘IS_A’ relationship (Figure 1), such as MeSH, DO, etc. Among them, DO is an ontology to organize vocabularies around diseases themselves [21]. And it integrates disease and medical vocabularies through extensive cross mapping [21]. Other vocabularies often include not only diseases themselves, but also terms of pathology, anatomical, etc. For example, MeSH is a more comprehensive ontology that has been classified as 16 categories. In these categories, only categories C and F03 define terms around disease. However, not all the terms in these categories are named for diseases themselves, such as pain (D010146). Furthermore, DO has been validated to be suitable for calculating disease similarity [11], [13], [28]. Therefore, we choose DO as disease terminology to describe disease terms for calculating disease similarity.

Function-based methods calculate disease similarity according to functional associations between genes. Semantic-based methods exploit associations from ontologies and the number of disease-related genes to compute disease similarity. Obviously, not all associations between diseases are represented by the ontology, a part of them are reflected through functional associations among disease-related genes and vice versa. In this paper, a new method (SemFunSim) is proposed, which integrates semantic and gene functional association for measuring similarity between diseases.

## Materials and Methods

### Disease Ontology

DO [21] (Table 1) contains 8,632 disease terms and 7,232 ‘IS_A’ relationships among diseases. The directed acyclic graph (DAG) of DO represents terms linked by ‘IS_A’ relationship, of which a node represents a DO term and an edge represents an ‘IS_A’ relationship between diseases. Figure 1 shows a sub-graph of the DAG starting from the specific DO term ‘Cutaneous lupus erythematosus (DOID:0050169)’ and ending at the root term of DO.

**Table 1:** Data sources used for measuring disease similarity.

Data source	Web site (Date of download)
DO	https://diseaseontology.svn.sourceforge.net/svnroot/diseaseontology/trunk/ (Apr 2013)
SIDD	http://mlg.hit.edu.cn/SIDD (Jul 2013)
CTD	http://ctdbase.org/downloads/;jsessionid=71BC29A1A48AD67BADA2E2C4FC9625F3 (Apr 2013)
HumanNet	http://www.functionalnet.org/humannet/download.html (Jul 2013)
GO	http://www.geneontology.org/GO.downloads.ontology.shtml (Jul 2013)
GOA	http://www.geneontology.org/GO.downloads.annotations.shtml (Jul 2013)
MimMiner	http://www.cmbi.ru.nl/MimMiner/suppl.html (Feb 2014)

### HumanNet and disease-related gene set

We accessed functional interactions of genes from HumanNet [29], which is an extended gene functional interaction network for Homo sapiens. Multiple distinct lines of evidence, spanning human mRNA co-expression, protein-protein interaction, protein complex, and comparative genomics data sets, in combination with similar lines of evidence from orthologs in yeast, fly and worm are comprehensively analyzed for the network using a probabilistic method [29]. This function network contains 476,399 interactions among 16,243 genes (Table 1).

Disease-related gene sets are from SIDD [30], which integrates five disease-related gene databases: GeneRIF [31], Online Mendelian Inheritance in Man (OMIM) [32], comparative toxicogenomics database (CTD) [33], genetic association database (GAD) [34], and SpliceDisease [35]. In total, 2,817 diseases, 12,063 genes and 117,190 associations between them are involved (Dataset S1). The data sources were downloaded from the web in Jul 2013, and the detailed information is listed in Table 1. Gene names in these sources have been converted to HUGO Gene Nomenclature Committee (HGNC) approved gene symbols [36].

### Disease similarity

**Figure 2:**
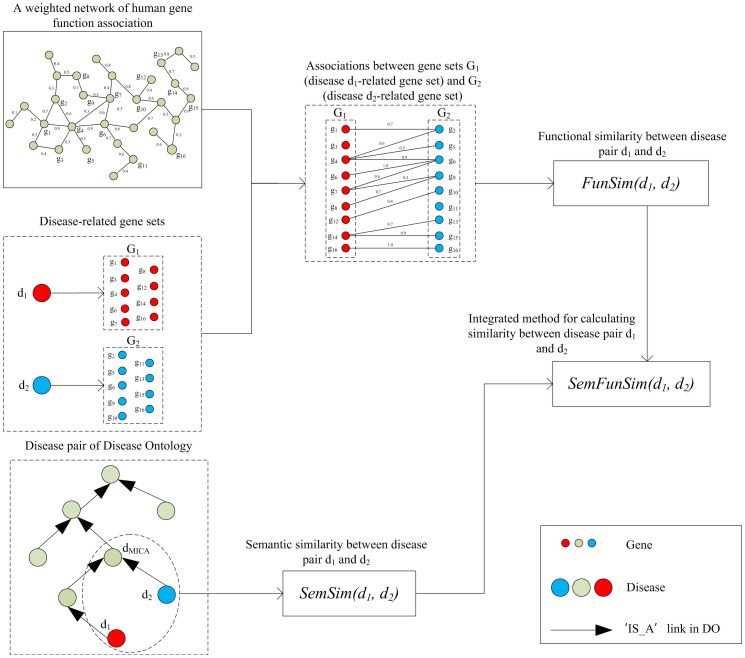
Overview of SemFunSim. d_1_, d_2_ are two diseases, and d_MICA_ is the MICA of d_1_ and d_2_. G_1_, G_2_ and G_MICA_ represent gene sets related to d_1_, d_2_ and d_MICA_, respectively.

Figure 2 gives an overview of SemFunSim. In the figure, d_1_ and d_2_ are two diseases from DO, and d_MICA_ is the MICA of d_1_ and d_2_. G_1_, G_2_ and G_MICA_ are gene sets related to d_1_, d_2_ and d_MICA_, respectively. First, a weighted network of human gene function association is used for calculating FunSim (functional similarity) between G_1_ and G_2_. Then, semantic associations from DO are used to calculate semantic similarity (SemSim) between diseases. Finally, FunSim and SemSim are integrated into SemFunSim.

#### Functional similarity between disease-related gene sets

Gene function networks are widely used to understand disease [29], [37]–[43]. We accessed the interactions of genes from HumanNet [29], which has been used to understand associations across three GO categories [44]. Each interaction of HumanNet has an associated log likelihood score (LLS) that measures the probability of a functional linkage between genes [29]. We normalized the associated LLS with equation 1.
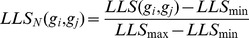
(1)where 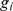 and 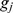 indicate the *i*th and *j*th gene, respectively. 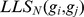 represents LLS between 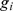 and 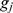 after normalization. 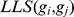 represents LLS between 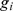 and 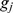. 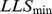 and 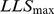 are the minimum LLS and the maximum LLS of HumanNet, respectively.

The functional similarity score between a pair of genes is defined as 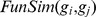: 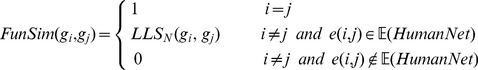
(2)


In equation 2, 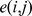 represents the interaction edge between gene pair 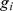 and 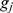. 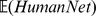 is a set which includes all the edges of HumanNet.

Then, we define the functional association between a gene 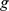 and a gene set 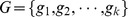 as 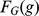, which is described in equation 3. 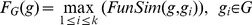
(3)where 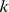 indicates the number of genes in 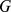, 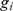 is the *i*th gene of 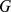.

Let a pair of gene sets 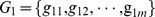 and 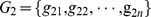 be related to diseases 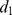 and 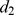, respectively. 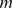 is the number of genes in 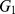, and 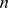 is the number of genes in 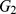. We define FunSim of 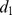 and 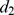 in equation 4 as follows. 
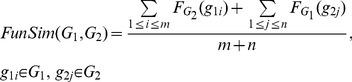
(4)


#### Semantic similarity based on Disease Ontology

We define semantic similarity between disease pair 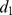 and 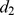 in equation 5. 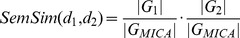
(5)where 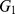 and 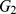 are gene sets related to 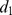 and 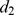, respectively. 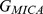 is gene set related to 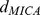, which represents the MICA of 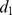 and 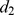 in the DAG of DO. 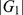, 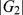, and 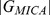 represent the number of genes in 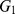, 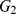 and 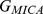, respectively.

#### Similarity between disease pair by SemFunSim

The similarity between disease pair 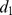 and 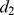 is defined in equation 6. 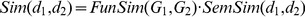
(6)where 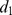 and 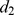 are two diseases of DO. 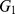 and 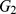 are gene sets related to 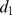 and 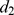, respectively.

A threshold for significant similarity of the 916 diseases with potential therapeutic chemicals (PTCs) in CTD is defined based on randomized data as follows. First, the 916 disease names in the DAG of DO were randomly shuffled, and the hierarchical structure remained the same as the original DO. Next, gene names in HumanNet were randomly shuffled, and the network topology remained the same as the original HumanNet. Then, the similarity scores for pairs of these 916 diseases were computed by SemFunSim based on the randomized data. The experiment was iterated 1000 times. Finally, we calculate the false discovery rate (FDR) over all pairs according to equation 7. 
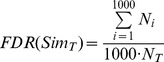
(7)where 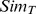 represents a similarity score, 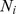 indicates the number of hits in the *i*th permutation with the similarity score > 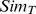, and 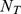 is the number of hits in the real case with the similarity score ≥ 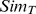.

## Results and Discussion

### Validation of disease similarity methods on benchmark set

**Figure 3:**
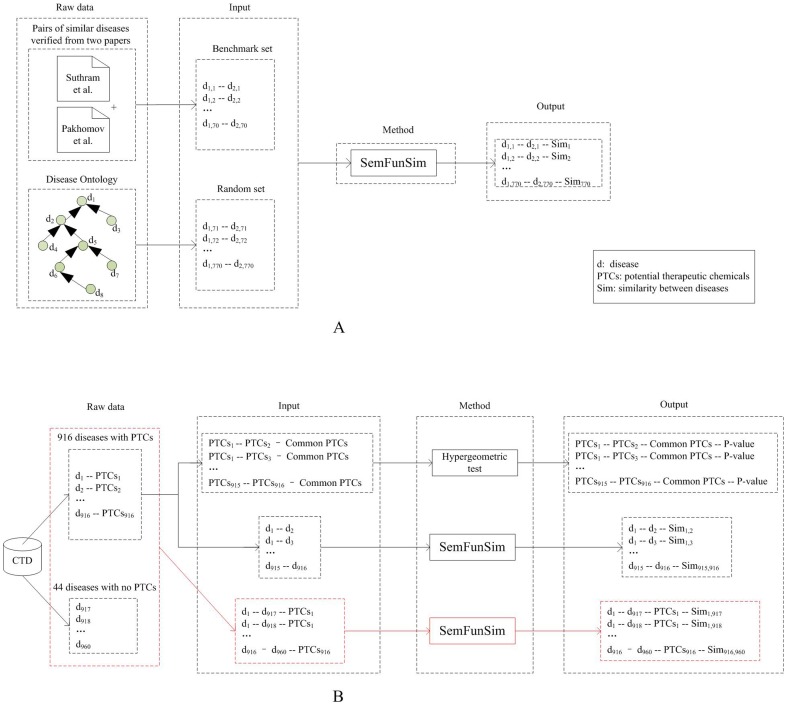
The process of validation. A. The similarities of disease pairs from the benchmark set and 100 random sets were calculated by SemFunSim, FunSim, Resnik, Wang, BOG, and PSB. B. The similarities of all the disease pairs between 916 diseases with PTCs in CTD were measured by SemFunSim, FunSim, Resnik, Wang, BOG, and PSB. In addition, the similarities of all the disease pairs between these 916 diseases with PTCs and 44 diseases without PTCs in CTD were computed by SemFunSim.

We calculated similarities of disease pairs on a benchmark set and another 100 random sets. The performance of SemFunSim was accessed by drawing a receiver operating characteristic (ROC) [45] curve. In Figure 3A, two types of disease pair sets are introduced as input in the validation process. On one hand, two manually checked datasets [12], [13], [46] of disease pairs with high similarity were integrated into a benchmark set. One dataset was obtained from diseases analyzed in the study by Suthram et al [12]. Disease pairs of the dataset were marked as similar after validation from literature by Mathur et al [13]. The other dataset was derived from the judgment of medical residents for semantic similarity, and pairs of similar diseases were extracted by Pakhomov et al [46]. In total, 47 diseases and 70 pairs of these two disease pair datasets were merged as the benchmark set (Dataset S2). On the other hand, each random set contains 700 disease pairs randomly selected from DO.

In order to further test the performance of the proposed method, SemFunSim was compared with disease similarity methods including Resnik [17], Wang [22], BOG [11], and PSB [13]. During the experiment, the parameters of these methods are selected according to the original paper.

**Figure 4:**
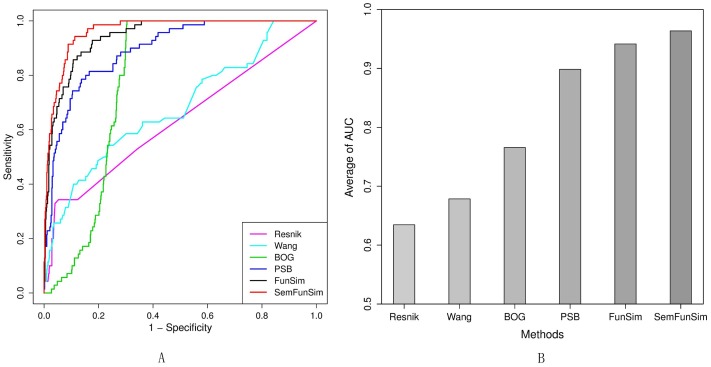
AUC analysis of the benchmark set and random sets. A. ROC curves for the experimental results on the benchmark set and a random set. It shows 1-specificity versus sensitivity of each method for calculating the similarities of disease pairs. B. Average of AUC for 100 permutations.

Similarities of disease pairs of the benchmark set and a random set were calculated by SemFunSim. We examined whether similarities of disease pairs of benchmark set could be prioritized in the top to produce an ROC curve. In Figure 4A, the area under the ROC curve (AUC) of each method is listed as follows, Resnik (63.14%), Wang (68.04%), BOG (78.10%), PSB (89.52%), and SemFunSim (96.36%). FunSim is part of SemFunSim, and has an AUC of 94.37%. The AUC shows that Wang et al.'s method is a little better than Resnik's method. The BOG method has the worst performance among function-based methods. When linking genes based on the GO biological process category [14] by the PSB method, the result has been improved significantly. Although the PSB method shows a very high AUC, FunSim still improves the results of the PSB method by about 5%. After integrating gene functional and semantic association, the SemFunSim method improves the performance further to nearly 100%. This experiment was iterated 100 times by calculating similarities of 100 random sets and the benchmark set. In Figure 4B, the average AUC of the 100 permutations is 0.6345, 0.6784, 0.7657, 0.8984, 0.9415, and 0.9637 for Resnik, Wang, BOG, PSB, FunSim, and SemFunSim, respectively. The result is consistent with Figure 4A.

Currently, functionally relevant gene associations can be defined in multiple ways (e.g. annotations for co-expression [25], protein-protein interaction [26], etc.). However, only one or two types of gene functional associations have been used to calculate the similarity by BOG and PSB [11], [13]. FunSim was designed for calculating disease similarity based on a comprehensive weighted gene functional association network. In Figure 4, the AUC of FunSim is higher than BOG and PSB. The results show that comprehensive gene functional association is suitable for calculating disease similarity.

Among the five methods, Resnik's method used the IC of the MICA to calculate similarity between diseases. A few disease pairs of the benchmark set have only one common ancestor node, consequently the similarities of these diseases are zero according to Resnik (File S1). For example, the similarity between disease pair ‘diabetes mellitus (DOID:9351)’ and ‘Alzheimer's Disease (DOID:10652)’ is zero (File S1), because the MICA of these two diseases is the root node of DO (Figure S1), and the IC of the root node is zero. To avoid this problem for pairs of similar diseases with only one common ancestor, the IC is not used for measuring disease similarity in SemSim. The ROC curves in Figure 4A show clearly that SemFunSim has the highest AUC, which validates that the integrated semantic association helps to enhance the true positive rate and reduce the false positive rate.

### Assessment of disease similarity by means of common therapeutic compounds

CTD (Table 1) [33] was introduced to compare PTCs for diseases (Figure 3B). CTD not only documents disease-related genes, but also documents disease-related markers and potential therapeutic compounds for diseases. Only potential therapeutic compounds for diseases were extracted as PTCs. In a previous study, disease terms of CTD were integrated with DO [30]. After extracting PTCs for diseases from CTD, 916 diseases, 3,522 chemicals and 11,134 associations were retained (Dataset S3). In addition, 44 diseases without PTCs in CTD were also kept.

**Figure 5:**
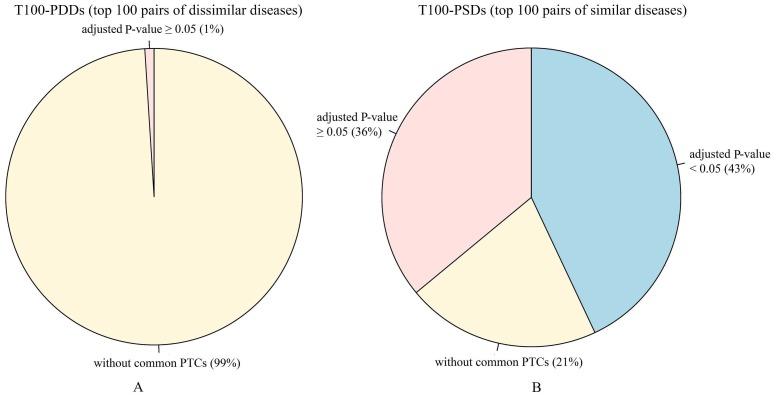
The number of pairs of diseases identified using SemFunSim with common PTCs. A. The number of pairs of the T100-PDDs with common PTCs. B. The number of pairs of the T100-PSDs with common PTCs. The yellow area represents the number of pairs without common PTCs. The pink area indicates the number of pairs with common PTCs and adjusted P-value ≥0.05. The light blue area represents the number of pairs with common PTCs and adjusted P-value <0.05.

In order to illustrate the point that similar diseases can often be treated with similar drugs [9], [47]–[49], PTCs for the top 100 pairs of similar diseases (T100-PSDs) and top 100 pairs of dissimilar diseases (T100-PDDs) (Dataset S4) identified using SemFunSim were compared. We counted the number of pairs with common PTCs and used a hypergeometric test to calculate the P-value for common PTCs for each pair of diseases. The P-value was adjusted by FDR [50]. There are 419,070 pairs between these 916 diseases. 1,251 pairs of them can be linked to each other by an ‘IS_A’ relationship of DO, which were not compared for avoiding diseases with common PTCs caused by the inclusion relationship. The results of the comparison are shown in Figure 5. 79 pairs of the T100-PSDs can be treated with common PTCs and 43 pairs have an adjusted P-value <0.05. In comparison, only 1 pair of the T100-PDDs can be treated with common PTCs and no pair has an adjusted P-value <0.05. The results show that the higher the similarity of a pair of diseases, the more likely they can be treated with common PTCs. Therefore, SemFunSim confirms the assumption that similar diseases can often be treated with similar drugs [9], [47]–[49].

**Figure 6:**
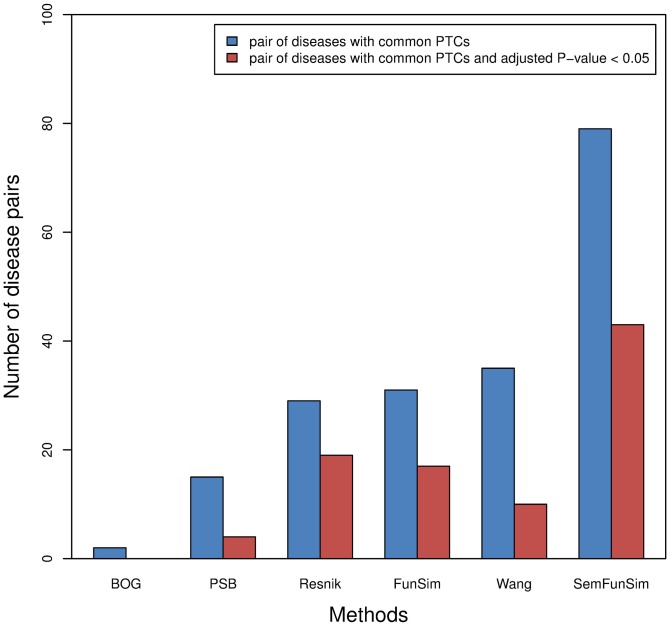
The number of pairs of similar diseases identified using the five methods with common PTCs. Blue bar indicates the number of pairs with common PTCs. Red bar represents the number of pairs with common PTCs and adjusted P-value <0.05.

We further compared the PTCs for the T100-PSDs identified by the five methods (Dataset S5). The results are shown in Figure 6. 2, 15, 29, 31, 35, 79 pairs of the T100-PSDs identified by BOG, PSB, Resnik, FunSim, Wang and SemFunSim respectively can be treated with common PTCs, and 0, 4, 19, 17, 10, 43 pairs of the T100-PSDs identified by BOG, PSB, Resnik, FunSim, Wang and SemFunSim respectively have an adjusted P-value <0.05. FunSim is part of SemFunSim and is designed by considering comprehensive gene functional association. It identifies a higher number of pairs of diseases with common PTCs than BOG and PSB. It shows that disease similarity calculated by comprehensive gene function association is appropriate for taking advantage of the fact that similar diseases can often be treated with similar drugs [9], [47]–[49]. The SemFunSim method identifies more than twice the number of pairs with common PTCs than the other methods. This confirms that SemFunSim is very suitable for the task.

The same test was applied to the top 500 pairs of similar diseases (T500-PSDs) and the top 1000 pairs of similar diseases (T1000-PSDs) identified by the five methods (Dataset S5). The results are shown in Table S1. In the table, 57, 247, 281, 308, 457, and 556 pairs of the T1000-PSDs identified by BOG, Resnik, Wang, PSB, FunSim, and SemFunSim respectively can be treated with common PTCs. And 9, 99, 90, 104, 170, and 237 pairs of the T1000-PSDs identified by BOG, Resnik, Wang, PSB, FunSim, and SemFunSim respectively have an adjusted P-value <0.05. The performance of Resnik, FunSim and Wang appears to be roughly the same in the T100-PSDs. After comparing more pairs of similar diseases (T500-PSDs and T1000-PSDs), FunSim performs better than Resnik and Wang (Table S1). The experimental results in Table S1 show that SemFunSim has an advantage over other compared methods.

Using random permutations of the functional gene network and the 916 diseases with PTCs in CTD, as described in the Methods section, we defined thresholds for significant similarity. We found that 448 pairs of diseases have a similarity score above 0.06060 at an FDR less than 0.05, and 6,981 pairs of diseases have a similarity score above 0.00111 at an FDR less than 0.10. The FDRs for pairs of diseases with the similarity score above 0.00111 are listed in Dataset S6. The threshold can be defined as 0.06060 (FDR <0.05). In addition, researchers can also adjust the threshold to validate more disease pairs, such as 0.00111 (FDR <0.10).

In an early study, van Driel et al. [51] developed a tool (MimMiner), which was extensively used to calculate similarity between phenotype terms from OMIM [52]. We obtained the similarity score between 5,080 OMIM phenotype records from MimMiner (Table 1). As mentioned before, CTD includes 916 diseases with PTCs. 127 common diseases between the 5,080 OMIM phenotype records and these 916 diseases (Dataset S7) were found through DO's extensive cross mapping [21]. Then, SemFunSim and MimMiner were compared on the basis of these 127 diseases.

**Figure 7:**
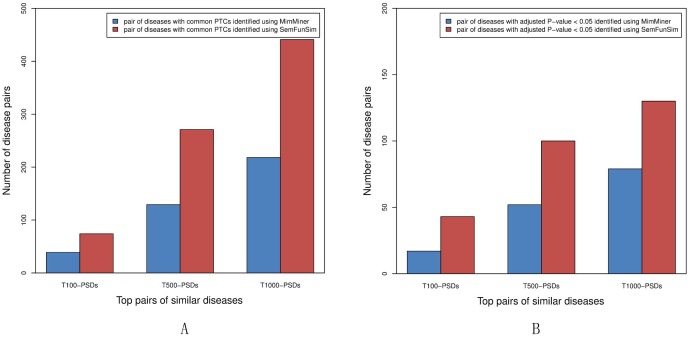
The number of pairs of similar diseases identified using MimMiner and SemFunSim with common PTCs. A. The number of pairs of the top pairs of similar diseases with common PTCs. The red bar represents the number of pairs with common PTCs measured by SemFunSim. The blue bar indicates the number of pairs with common PTCs measured by MimMiner. B. The number of pairs of the top pairs of similar diseases with common PTCs and adjusted P-value <0.05. The red bar represents the number of pairs with common PTCs and adjusted P-value <0.05 measured by SemFunSim. The blue bar indicates the number of pairs with common PTCs and adjusted P-value <0.05 measured by MimMiner.

The result of the comparison is shown in Figure 7. 39, 129, and 218 pairs of the T100-PSDs, T500-PSDs, and T1000-PSDs identified by MimMiner respectively can be treated with common PTCs. And 17, 52, and 79 pairs of the T100-PSDs, T500-PSDs, and T1000-PSDs respectively have an adjusted P-value <0.05. In comparison, 74, 271, and 441 pairs of the T100-PSDs, T500-PSDs, and T1000-PSDs identified by SemFunSim respectively can be treated with common PTCs. And 43, 100, and 130 pairs of the T100-PSDs, T500-PSDs, and T1000-PSDs respectively have an adjusted P-value <0.05. Result shows that similar diseases identified using SemFunSim are very likely to be treated with common drugs.

We further compared MimMiner and SemFunSim based on their thresholds. 53 pairs of the 127 common diseases identified by MimMiner have a similarity >0.4 (threshold of MimMiner) (Dataset S7). 23 (43.4%—23/53) of them can be treated with common PTCs, and 9 (17.0%—9/53) have an adjusted P-value <0.05. In comparison, 107 pairs of the 127 diseases identified by SemFunSim have a similarity >0.00111 (threshold of SemFunSim) (Dataset S7). 78 (72.9%—78/107) of them can be treated with common PTCs, and 44 (41.1%—44/107) have an adjusted P-value <0.05. The experiment results based on these 127 diseases show that SemFunSim's performance in measuring disease similarity is better than MimMiner's.

### Prediction of novel therapeutic applications of known compounds

SemFunSim was used to find PTCs for 44 diseases without PTCs in CTD. First, as shown in Figure 3B, we calculated similarities of 40,304 pairs between these 44 diseases and 916 diseases with PTCs in CTD (Dataset S8). In order to avoid diseases with common PTCs caused by the inclusion relationship, 64 of the 40,304 pairs which can be linked with each other by an ‘IS_A’ relationship of DO were not included. Each pair of the 40,240 pairs includes one disease without PTCs in CTD and one disease with PTCs in CTD. The top 20 pairs of similar diseases (T20-PSDs) (Table 2) contain 12 diseases without PTCs in CTD. Then, we searched PubMed to find PTCs for these 12 diseases. According to the idea that similar diseases can often be treated with similar drugs, the PTCs for one disease in pair of similar diseases can be used as a reference for the other without PTCs. For example, ‘systemic scleroderma’ is similar with ‘polymyalgia rheumatica’, 11 PTCs for the former are documented in CTD. We searched from PubMed for finding associations between these 11 PTCs and ‘polymyalgia rheumatica’. And we found that four of them were also PTCs for ‘polymyalgia rheumatica’, such as azathioprine [53], Methylprednisolone [54], Prednisolone [55] and Prednisone [56]. Finally, 6 of these 12 diseases from the T20-PSDs can be treated with PTCs confirmed by literature. The detailed results are listed in Table 3, which indicate that SemFunSim is an effective method to find PTCs for diseases.

**Table 2:** Top 20 pairs of similar diseases.

Order	Diseases with PTCs in CTD	Diseases without PTCs in CTD	Similarities
1	Liver Cirrhosis	Hepatopulmonary syndrome	0.03460
2	agranulocytosis	lymphopenia	0.01665
3	neutropenia	lymphopenia	0.01566
4	macroglobulinemia	alpha 1-antitrypsin deficiency	0.01424
5	hepatitis	hepatopulmonary syndrome	0.00887
6	wilson disease	hemochromatosis	0.00862
7	systemic scleroderma	polymyalgia rheumatica	0.00717
8	drug-induced hepatitis	hepatopulmonary syndrome	0.00710
9	myasthenia gravis	lambert-eaton myasthenic syndrome	0.00644
10	dilated cardiomyopathy	restrictive cardiomyopathy	0.00643
11	sarcoidosis	cryoglobulinemia	0.00607
12	berylliosis	asbestosis	0.00600
13	berylliosis	extrinsic allergic alveolitis	0.00575
14	intestinal disease	hepatopulmonary syndrome	0.00564
15	placenta disease	bacterial vaginosis	0.00499
16	hyperthyroidism	congenital hypothyroidism	0.00461
17	biliary tract disease	hepatopulmonary syndrome	0.00454
18	bile duct disease	hepatopulmonary syndrome	0.00452
19	inflammatory bowel disease	hepatopulmonary syndrome	0.00447
20	primary biliary cirrhosis	hepatopulmonary syndrome	0.00421

The first column is the descending order number of similarity between diseases. The second column represents diseases with PTCs in CTD. The third column indicates diseases without PTCs in CTD. The fourth column represents the similarities between pairs of diseases in the second and third columns.

**Table 3:** Associations between PTCs and diseases retrieved from PubMed.

Order	PTCs	Diseases with PTC in CTD	Diseases without PTC in CTD	Similarities	PMIDs
1	Pentoxifylline	Liver Cirrhosis	Hepatopulmonary syndrome	0.03460	23002364 [57]
5	Acetylcysteine	hepatitis	hepatopulmonary syndrome	0.00887	18341514 [58]
7	Azathioprine	systemic scleroderma	polymyalgia rheumatica	0.00717	2750226 [53]
7	Methylprednisolone	systemic scleroderma	polymyalgia rheumatica	0.00717	1768166 [54]
7	Prednisolone	systemic scleroderma	polymyalgia rheumatica	0.00717	8523341 [55]
7	Prednisone	systemic scleroderma	polymyalgia rheumatica	0.00717	15466766 [56]
8	Pentoxifylline	drug-induced hepatitis	hepatopulmonary syndrome	0.00710	23002364 [57]
9	Prednisolone	myasthenia gravis	lambert-eaton myasthenic syndrome	0.00644	10555101 [59]
11	Methylprednisolone	sarcoidosis	cryoglobulinemia	0.00607	6851261 [60]
13	Prednisone	berylliosis	extrinsic allergic alveolitis	0.00575	9489437 [61]
16	Methimazole	hyperthyroidism	congenital hypothyroidism	0.00461	22672871 [62]
19	Acetylcysteine	inflammatory bowel disease	hepatopulmonary syndrome	0.00447	18341514 [58]

The first column is the descending order number of similarity between diseases. PTCs (in the second column) for diseases (in the third column) are documented in CTD. The fourth column represents diseases without PTCs in CTD. The fifth column indicates the similarities between pairs of diseases in the third and fourth column. The sixth column is the PubMed IDs that record the associations between PTCs (in the second column) and diseases (in the fourth column).

## Conclusions

In this article, we devise an algorithm (SemFunSim) to measure disease similarity by integrating FunSim and SemSim effectively. Experimental evaluation was performed on the benchmark set and 100 random sets from DO. The high average AUC (96.37%) shows that SemFunSim achieves a high true positive rate and a low false positive rate.

SemFunSim is in agreement with the notion that similar diseases can often be treated with similar drugs [9], [47]–[49]. SemFunSim not only helps to understand associations between diseases, but also provides an effective way to predict PTCs for diseases. We found associations between diseases and PTCs that were not documented in CTD using SemFunSim (Table 3).

## Supporting Information

File S1 **Description of IC and MICA.**
(DOCX)

Figure S1 **A sub-graph of the DAG for DO term ‘pick's disease (DOID:11870)’, ‘Alzheimer's Disease (DOID:10652)’ and ‘Diabetes mellitus (DOID:9351)’.** The arrow symbol represents an ‘IS_A’ link of DO. For example, “Alzheimer's Disease (DOID:10652)” is linked to “Dementia (DOID:1307)” by an ‘IS_A’ relationship.
(DOCX)

Table S1 **The number of pairs of the T500-PSDs and T1000-PSDs measured by the five methods with common PTCs.**
(DOCX)

Dataset S1 **Associations between diseases and genes.**
(XLSX)

Dataset S2 **Benchmark set.**
(XLSX)

Dataset S3 **Associations between diseases and PTCs.**
(XLSX)

Dataset S4 **The T100-PSDs and T100-PDDs measured by SemFunSim.** 916 diseases can be treated with PTCs in CTD. The T100-PSDs and T100-PDDs between these 916 diseases were identified by SemFunSim. The disease pairs, the number of common PTCs between diseases, and adjusted P-values are listed.
(XLSX)

Dataset S5 **The T1000-PSDs measured by the five methods.** 916 diseases can be treated with PTCs in CTD. The T1000-PSDs between these 916 diseases were accessed by SemFunSim, FunSim, Wang, Resnik, PSB, and BOG. The disease pairs, the number of common PTCs between diseases, and adjusted P-values are listed.
(XLSX)

Dataset S6 **FDRs for the similarity scores.** The FDRs for pairs of diseases with the similarity score above 0.00111 are listed.
(XLSX)

Dataset S7 **The T1000-PSDs measured by MimMiner and SemFunSim.** The T1000-PSDs were identified using MimMiner and SemFunSim. The disease pairs, the number of common PTCs between diseases, and adjusted P-values are listed. In addition, 127 DO terms and their extensive cross-referenced OMIM phenotype records are listed.
(XLSX)

Dataset S8 **Similarities between 44 diseases without PTCs and 916 diseases with PTCs in CTD measured by SemFunSim.** We calculated the similarities between 44 diseases (without PTCs in CTD) and 916 diseases (with PTCs in CTD) based on SemFunSim. These 44 diseases without PTCs in CTD and the similarities are listed.
(XLSX)
