# Supplementary material for: SemFunSim: A New Method for Measuring Disease Similarity by Integrating Semantic and Gene Functional Association
Source: PLoS One. 2014 Jun 16;9(6):e99415. doi: 10.1371/journal.pone.0099415 (PMC4059643; doi:10.1371/journal.pone.0099415)
Supplement: File S1 — Description of IC and MICA. (DOCX) [file pone.0099415.s001.docx]

## Information content

The IC (information content) of a term of Disease Ontology (DO) [[1](#_ENREF_1)] is defined as the negative log likelihood [[2](#_ENREF_2)] as follows (equation S1):

$IC=-\log p(d)$ (S1)

where $d$ is a disease term of DO, is the number of genes related to $d$ divided by the total number of genes related to DO.

Because the related genes of descendant disease terms are also related to ancestor disease terms [[3](#_ENREF_3)], the number of genes related to the root term “Disease (DOID:4)” equals the total number of genes related to DO.

Figure S1 gives a sub-graph of the directed acyclic graph (DAG) for DO term ‘pick’s disease (DOID:11870)’, ‘Alzheimer's Disease (DOID:10652)’ and ‘Diabetes mellitus (DOID:9351)’. According to Figure S1, the IC of disease ‘pick’s disease (DOID:11870)’ is as follows (equation S2):

$IC_{DOID:11870}=-\log\frac{|G_{DOID:11870}|}{|G_{DOID:4}|}$ (S2)

where $IC_{DOID:11870}$ represents the IC of disease “pick’s disease (DOID:11870)”, $G_{DOID:11870}$ is related gene set of disease “pick’s disease (DOID:11870)”, $|G_{DOID:11870}|$ represents the number of genes in $G_{DOID:11870}$, $G_{DOID:4}$ is related gene set of disease “disease (DOID:4)”, and $|G_{DOID:4}|$ represents the number of genes in $G_{DOID:4}$. It is easy to see that the IC of the root term is zero.

## Most informative common ancestor

The MICA (most informative common ancestor) means the ancestor that has the maximum IC among all the common ancestors between terms of ontology [[2](#_ENREF_2)].

In Figure S1, there are five common ancestors including “Neurodegenerative disease (DOID:1289)”, “Central nervous system disease (DOID:331)”, “Nervous system disease (DOID:863)”, “Disease of anatomical entity (DOID:7)”, and “Disease (DOID:4)” between two diseases “pick’s disease (DOID:11870)” and “tauopathy (DOID:680)”. Obviously, the MICA of these two diseases is “Neurodegenerative disease (DOID:1289)”.

## Disease similarity by Resnik

According to Resnik’s method [[2](#_ENREF_2)], similarity between a pair of diseases is defined as follows (equation S3):

$Sim(d_{1},d{}_{2})=-\log p(d_{MICA})$ (S3)

where $Sim(d_{1},d{}_{2})$ represents similarity between a pair of diseases $d_{1}$ and $d_{2}$, $d_{MICA}$ indicates the MICA of $d_{1}$ and $d_{2}$.

The root node is the ancestor node of all other nodes. Therefore, if a pair of diseases has only one common ancestor node, the common ancestor node must be the root node. Correspondingly, the similarity of the disease pair by Resnik is zero according to equation S1 and equation S3. As shown in Figure S1, there is only one common ancestor “Disease (DOID:4)” between two diseases “Alzheimer's Disease (DOID:10652)” and “Diabetes mellitus (DOID:9351)”. Then, the similarity between these two diseases is zero based on equation S3.

## References

1. Schriml LM, Arze C, Nadendla S, Chang YW, Mazaitis M, et al. (2012) Disease Ontology: a backbone for disease semantic integration. Nucleic Acids Res 40: D940-946.

2. Resnik P. Using information content to evaluate semantic similarity in a taxonomy; 1995. Proceedings of the 14th international joint conference on artificial intelligence. Morgan Kaufmann Publishers Inc. pp. 448-453.

3. Smith B, Ceusters W, Klagges B, Kohler J, Kumar A, et al. (2005) Relations in biomedical ontologies. Genome Biol 6: R46.
