# Supplementary material for: SemFunSim: A New Method for Measuring Disease Similarity by Integrating Semantic and Gene Functional Association
Source: PLoS One. 2014 Jun 16;9(6):e99415. doi: 10.1371/journal.pone.0099415 (PMC4059643; doi:10.1371/journal.pone.0099415)

## Figure S1 (Supplementary Figure 1). A sub-graph of the DAG for DO terms ‘pick’s disease (DOID:11870)’, ‘Alzheimer's Disease (DOID:10652)’ and ‘Diabetes mellitus (DOID:9351)’.

The arrow symbol represents an ‘IS_A’ link of DO. For example, “Alzheimer's Disease (DOID:10652)” is linked to “Dementia (DOID:1307)” by an ‘IS_A’ relationship.


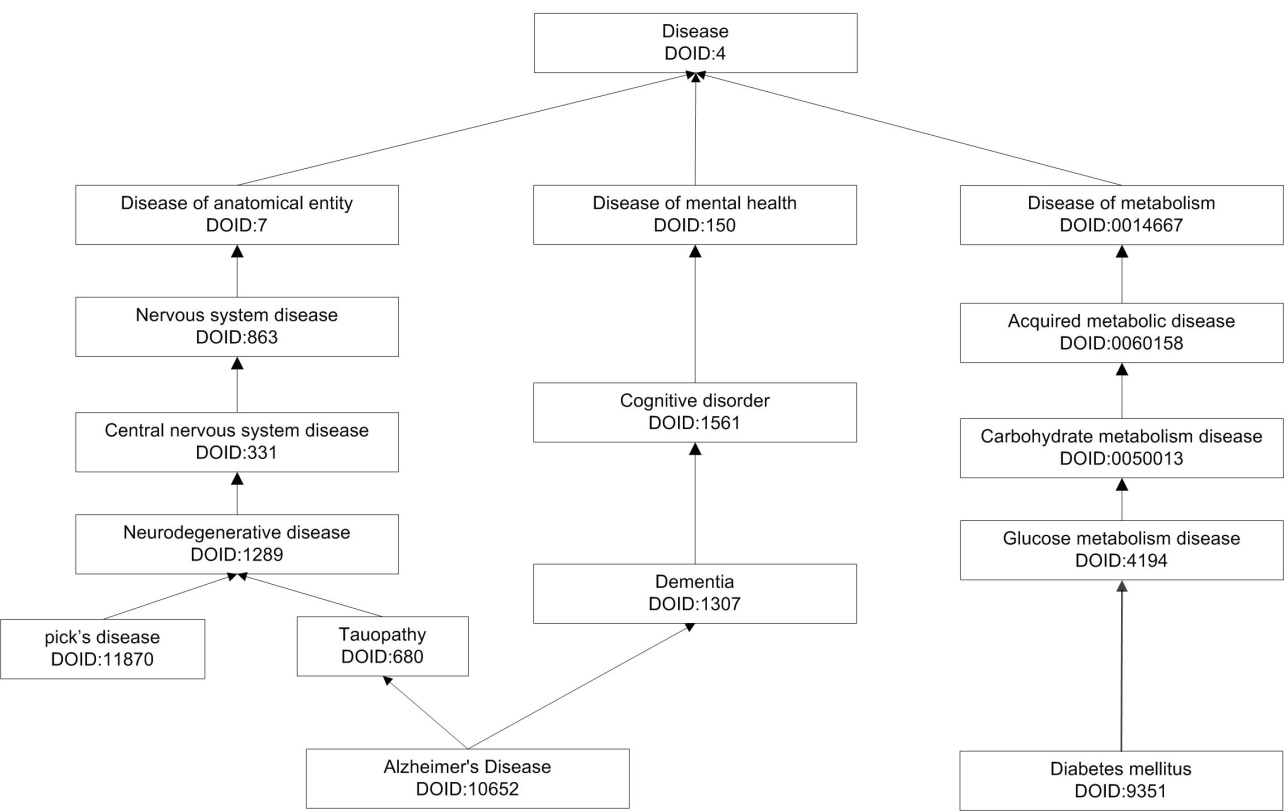

Supplement: Figure S1 — A sub-graph of the DAG for DO term ‘pick's disease (DOID:11870)’, ‘Alzheimer's Disease (DOID:10652)’ and ‘Diabetes mellitus (DOID:9351)’. The arrow symbol represents an ‘IS_A’ link of DO. For example, “Alzheimer's Disease (DOID:10652)” is linked to “Dementia (DOID:1307)” by an ‘IS_A’ relationship. (DOCX) [file pone.0099415.s002.docx]
