# Supplementary material for: SemFunSim: A New Method for Measuring Disease Similarity by Integrating Semantic and Gene Functional Association
Source: PLoS One. 2014 Jun 16;9(6):e99415. doi: 10.1371/journal.pone.0099415 (PMC4059643; doi:10.1371/journal.pone.0099415)
Supplement: Table S1 — The number of pairs of the T500-PSDs and T1000-PSDs measured by the five methods with common PTCs. (DOCX) [file pone.0099415.s003.docx]

## Table S1 (Supplementary Table 1). The number of pairs of the T500-PSDs and T1000-PSDs measured by five methods with common PTCs.

The first column lists methods. The second column is the number of disease pairs with common PTCs of the T500-PSDs and T1000-PSDs. The third column represents the number of disease pairs of the T500-PSDs and T1000-PSDs with common PTCs and adjusted P-value < 0.05.

| Method | Number of disease pairs with common PTCs | | Number of disease pairs with common PTCs and adjusted P-value < 0.05 | |
| --- | --- | --- | --- | --- |
|  | T500-PSDs | T1000-PSDs | T500-PSDs | T1000-PSDs |
| BOG | 32 | 57 | 3 | 9 |
| Resnik | 154 | 247 | 65 | 99 |
| Wang | 173 | 281 | 65 | 90 |
| PSB | 140 | 308 | 45 | 104 |
| FunSim | 215 | 457 | 105 | 170 |
| SemFunSim | 312 | 556 | 147 | 237 |
